# Prognostic Impact of mRNA Expression Levels of HER1–4 (ERBB1–4) in Patients with Locally Advanced Rectal Cancer

**DOI:** 10.1155/2016/3481578

**Published:** 2016-08-16

**Authors:** Melanie Kripp, Kirsten Merx, Ralph Markus Wirtz, Timo Gaiser, Sebastian Eidt, Juliana Schwaab, Stefan Post, Frederik Wenz, Andreas Hochhaus, Ralf-Dieter Hofheinz, Philipp Erben

**Affiliations:** ^1^III. Medizinische Klinik, Universitätsmedizin Mannheim, 68167 Mannheim, Germany; ^2^Stratifyer Molecular Pathology GmbH, 50935 Köln, Germany; ^3^Pathologisches Institut, Universitätsmedizin Mannheim, 68167 Mannheim, Germany; ^4^Chirurgische Klinik, Universitätsmedizin Mannheim, 68167 Mannheim, Germany; ^5^Klinik für Strahlentherapie und Radioonkologie, Universitätsmedizin Mannheim, 68167 Mannheim, Germany; ^6^Abteilung Hämatologie/Onkologie, Universitätsklinikum Jena, 07747 Jena, Germany; ^7^Klinik für Urologie, Universitätsmedizin Mannheim, 68167 Mannheim, Germany

## Abstract

*Background*. No predictive or prognostic biomarker is available for patients with locally advanced rectal cancer (LARC) undergoing perioperative chemoradiotherapy (CRT). Members of the human epidermal growth factor receptor (HER) family of receptor tyrosine kinases EGFR (HER1,* ERBB1*), HER2 (*ERBB2*), HER3 (*ERBB3*), and HER4 (*ERBB4*) are therapeutic targets in several cancers. The analysis was performed to assess expression levels and study the potential prognostic impact for disease-free and overall survival in patients with LARC.* Patients and Methods*.* ERBB1–4 *mRNA expression and tumor proliferation using Ki-67 (*MKI67*) mRNA were evaluated using RT-quantitative PCR in paraffin-embedded tumor samples from 86 patients (median age: 63) treated with capecitabine or 5-fluorouracil-based CRT within a phase 3 clinical trial.* Results*. A positive correlation of HER4 and HER2, HER3 and HER2, and HER4 and HER3 with each other was observed. Patients with high mRNA expression of* ERBB1* (EGFR, HER1) had significantly increased risk for recurrence and death. Patients with high mRNA expression of* MKI67* had reduced risk for relapse.* Conclusion*. This analysis suggests a prognostic impact of both* ERBB1* and MKi67 mRNA expression in LARC patients treated with capecitabine or fluorouracil-based chemoradiotherapy.

## 1. Introduction

Rectal cancer is the fifth most common type of cancer in adults worldwide [1]. Although surgery may be curative in locally advanced disease, local recurrence and metastases occur despite complete resection. Until the late 1980s, the rate of local recurrence and distant metastases following curative surgery was about 30% [2]. The outcome of locally advanced rectal cancer (LARC) has significantly improved due to the combination of optimized surgical techniques, notably total mesorectal excision (TME) with neoadjuvant radio- and radiochemotherapy [3–5]. By using this treatment modality, 10-year cumulative local recurrence rate is generally below 10%. On the other hand, fluorouracil in conjunction with neoadjuvant long-term radiotherapy reduces local recurrences but does not prolong overall survival (OS) [6, 7].

Nowadays,* distant* metastases represent the most common type of treatment failure in rectal cancer indicating the need for optimized systemic medical treatment such as modifications of perioperative bolus 5-fluorouracil (5-FU) treatment. However, neither biomodulation of fluorouracil nor combinations with older cytostatic drugs have clearly proved advantage compared to bolus 5-FU [8]. Only administering 5-FU as continuous infusion during radiation led to improved survival and increased time to relapse [9]. Likewise, the addition of oxaliplatin to perioperative fluorouracil treatment has given diverging results in five clinical trials [10, 11].

Altogether, the identification of patients being at high risk for distant metastases still represents a major challenge to tailor the management of rectal cancer therapy. For patients with resected rectal cancer—in addition to the adequacy of surgical excision (evaluated by the circumferential resection margins)—the TNM classification is still the most reliable indicator of risk for systemic recurrence [12]. However, for patients scheduled to undergo neoadjuvant chemoradiotherapy* clinical* stratification (e.g., with MRI scan) has limitations especially in view of the inability to exactly predict the nodal status. Risk stratification based on molecular markers could provide better estimate of individual risk and tailored treatment. In this regard, especially molecular markers bearing the potential to serve as therapeutic targets for medical treatment are indispensable.

Several drugs are licensed or in clinical evaluation for the treatment of tumors expressing human epidermal growth factor tyrosine kinase receptors (EGFR). Besides EGFR, which is encoded by the* ERBB1* gene, the human epidermal growth factor receptor (HER) family of receptor tyrosine kinases includes three additional members: HER2 (*ERBB2*), HER3 (*ERBB3*), and HER4 (*ERBB4*). Activation of the HER signaling network has been shown to promote tumor invasion and metastasis both in vitro [13–16] and in vivo [17, 18].

Mutations and overexpression of HER family genes are frequently present in rectal cancer. For instance, a higher rate of HER2 amplification in high grade tumors was reported, and EGFR and HER3 mRNA expression was described to be associated with the occurrence of metastases in patients with LARC [19].

Here, we sought to evaluate (a) the mRNA expression of ERBB1–4 in pretreatment tumor tissue, (b) the correlation of expression of each receptor along with tumor cell proliferation using MKI67 mRNA expression in patients with LARC treated with 5-FU-based chemoradiotherapy, and (c) the impact of these markers on prognosis.

## 2. Material and Methods

*Patient Cohort*. Tumor tissue for this study stemmed from patients participating in a phase III clinical trial conducted at the University Hospital of Mannheim between 2002 and 2007. These patients had histologically confirmed LARC (adenocarcinoma, cT3-4, any N or cT2, N+). This noninferiority trial compared 5-FU with the oral 5-FU prodrug capecitabine for the perioperative treatment of LARC. Details of the study protocol and the results have been published previously [20]. In brief, the trial began in 2002 as an adjuvant trial comparing capecitabine-based chemoradiotherapy with fluorouracil-based chemoradiotherapy. Patients in the capecitabine group were scheduled to receive two cycles of capecitabine, followed by chemoradiotherapy (50.4 Gy plus capecitabine 1650 mg/m^2^), then three cycles of capecitabine. Patients in the fluorouracil group received two cycles of bolus fluorouracil followed by chemoradiotherapy (50.4 Gy plus infusional fluorouracil 225 mg/m^2^ daily), then two cycles of bolus fluorouracil. The protocol was amended in 2005, to allow a neoadjuvant cohort in which patients in the capecitabine group received chemoradiotherapy followed by TME surgery and five cycles of capecitabine and patients in the fluorouracil group received chemoradiotherapy (50.4 Gy plus infusional fluorouracil 1000 mg/m^2^ days 1–5 and 29–33) followed by surgery and four cycles of bolus fluorouracil. Patients were randomly assigned to treatment groups in a 1 : 1 ratio. The primary endpoint was overall survival. Noninferiority of capecitabine in terms of 5-year overall survival was tested. 392 patients were evaluable with a median follow-up of 52 months. Five-year overall survival in the capecitabine group was noninferior to that in the fluorouracil group (76% [95% CI 67–82] versus 67% [58–74]; *p* = 0.0004). Similar numbers of patients had local recurrences in each group 12 [6%] in the capecitabine group versus 14 [7%] in the fluorouracil group, *p* = 0.67.

All patients providing tumor tissue for the current analysis were treated at a single center (University Hospital Mannheim, University of Heidelberg).

Both the clinical study protocol and the molecular investigations reported here were approved by the local ethics committee.

### 2.1. RNA Isolation from Formalin-Fixed Paraffin-Embedded (FFPE) Tissue and Quantitative Reverse Transcription-Polymerase Chain Reaction (RT-qPCR) Assessment

Fixation of tumor specimens followed standard protocols, 10% buffered formalin for at least 8 hours. Hematoxylin-eosin (HE) stained sections were evaluated for pathological stage according to the 2002 TNM classification of the American Joint Committee on Cancer classification. For molecular analyses, HE slides were reevaluated by an experienced pathologist (S.E.) for confirmation of the presence of invasive tumor. Each case was macrodissected for an invasive tumor areal comprising at least 30% of tumor cells. One 10 *μ*m section was used for the isolation of RNA according to a fully automated, high-throughput extraction workflow which runs on an Xtract-XL liquid-handling robot (STRATIFYER Molecular Pathology GmbH, Cologne, Germany). The extraction solutions and chemicals are commercially available in Germany as part of the XTRAKT FFPE kit, which is based on magnetic bead technology (STRATIFYER) [21]. In brief, FFPE sections were solubilized and paraffin was melted by incubating with a lysis buffer in a Thermo-mixer. Tissue was lyzed with Proteinase K. The lysates were then admixed with germanium-coated magnetic particles in buffer-controlled conditions which enhance preferential attachment of nucleic acid molecules to the surface of the particles. Purification was carried out by means of 3 consecutive washing cycles involving magnetization, centrifugation, washing, and removal of the supernatant. Nucleic acids were eluted with 100 *μ*L elution buffer and treated with DNase I. The DNA-free RNA eluates were stored at −80°C until use.

One-step RT-qPCR was applied for the relative quantification of* EGFR (ERBB1)*,* ERBB2 (HER2)*,* ERBB3 (HER3)*,* ERBB4 (HER4),* and* MKI67* mRNA expression by using gene-specific TaqMan®-based assays as described [22]. Expression levels of the target genes as well as of the reference gene Calmodulin 2 (*CALM2*) were assessed in duplicate by RT-qPCR using the SuperScript III PLATINUM One-Step, quantitative RT-PCR System (Invitrogen, Karlsruhe, Germany) on a Stratagene Mx3005p (Agilent Technologies, Böblingen, Germany) with 30 min at 50°C and 2 min at 95°C followed by 40 cycles of 15 sec at 95°C and 30 sec at 60°C.

Forty cycles of nucleic acid amplification were applied and the cycle threshold (Cq) values of the target genes were identified. Cq values were normalized by subtracting the Cq value of the housekeeping gene* CALM2* from the Cq value of the target genes (ΔCq). RNA results were then reported as 40-ΔCq values which correlate proportionally with the mRNA expression level of the target genes. The quantity of RNA following isolation (yield) was determined by measuring* CALM2* expression as a surrogate marker for amplifiable mRNA. Samples with average* CALM2* Cq values <32 were considered to have sufficient RNA and were eligible for further analysis. Therefore 5 of the 106 extracted samples (success rate 95%) had an average* CALM2* CT value of ≥32 and were therefore excluded. CALM2 were selected as control gene based on molecular studies in breast cancer patients which showed a high stability of this gene [23]. The lengths of the amplicons detected by the EGFR (*ERBB1*)*, ERBB2, ERBB3, ERBB4,* and* MKI67 *assays were 93 bp, 61 bp, 81 bp, 75 bp, and 108 bp, respectively, with PCR efficiencies [*E* = 1(10-slope)] of 89.1, 97.2, 96.0, 92.0, and 99.7%, respectively. A commercially available human reference RNA (Stratagene qPCR Human Reference Total RNA, Agilent Technologies, Waldbronn, Germany) was used as positive control. No-template controls were assessed in parallel to exclude contamination.

Details of the primer/probe sets used for amplification of the target and reference genes are listed in Table 1.

### 2.2. Statistical Analysis

Overall survival (OS) was measured from the date of randomization until death from any cause. Surviving patients were censored at the date of last contact. Disease-free survival (DFS) was measured from the date of randomization until recurrence of tumor, secondary neoplasm, or death from any cause as described [20]. Time-to-event distributions were estimated using Kaplan-Meier analyses. Continuous variables were presented as medians with the corresponding range and categorical variables as frequencies with the respective percentages. Associations of marker genes with basic patient and tumor characteristics were examined using Fisher's exact test for categorical variables and Mann-Whitney test for continuous variables. Correlations between the target genes were calculated using Spearman's rank correlation coefficient (Rho). Univariate Cox regression analyses were performed to assess the relationship between markers and OS or DFS. The cut-offs with the highest predictive values for OS and DFS were estimated using the partitioning test. All *p* values were two-sided with *p* values < 0.05 indicating statistical significance. Statistical analyses were performed with SAS Jmp 10.0 (SAS Institute, Cary, NC, USA) and Graph Pad Prism software (Version 5, La Jolla, CA, USA). With regard to OS and DFS patient samples with pretherapeutic biopsies (*n* = 55) were analyzed in order to exclude a possible sampling error.

## 3. Results

### 3.1. Patients' Characteristics and mRNA Expression

**Figure 1:**
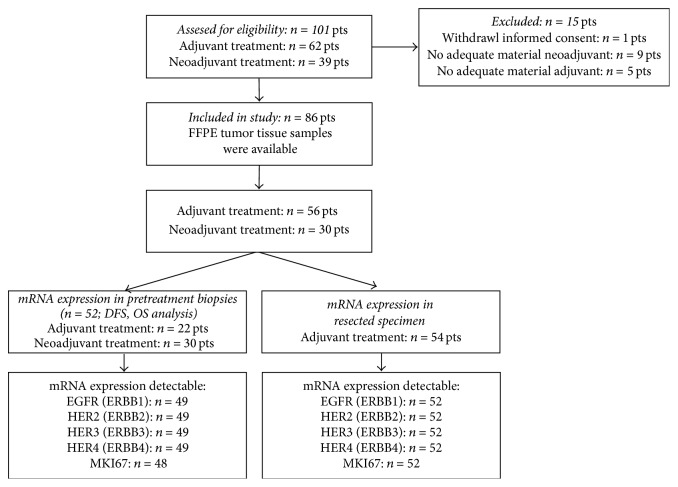
Participants and samples flow chart diagram.

**Figure 2:**
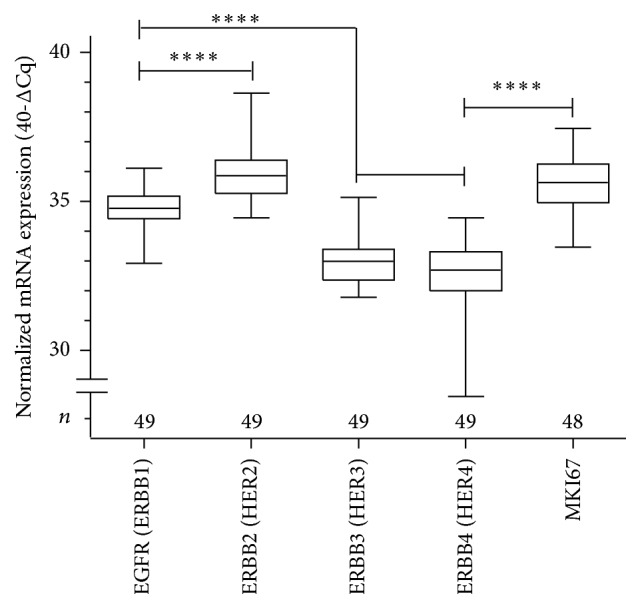
Distribution of mRNA expression values in analyzed biopsies. Normalized mRNA expression values (40-ΔCq) of RT-qPCR evaluated HER (ERBB) family marker genes and MKI67 are presented. Lowest expression levels were observed for ERBB3 and ERBB4 and highest expression for ERBB2 and MKI67 (box and whiskers plots ranging from minimum to maximum). Gene expression values were compared by two-way Mann-Whitney test (^*∗∗∗∗*^ *p* < 0.0001).

Tumor tissue samples were obtained from 86 patients (median age 63 years, range 44–83 years). A total of 52 biopsies and 54 resection specimens from these patients were analyzed as described in the “Material and Methods.” Only biopsies deriving prior to treatment were analyzed with regard to OS and DFS. Regarding these biopsies, 30/52 biopsies were derived from the additional neoadjuvant treated patient group and 22/52 biopsies from the solely adjuvant treated patients. Resection specimens were exclusively investigated in patients receiving solely adjuvant chemoradiotherapy to analyze a possible sample effect of biopsies and resected specimens (*n* = 54) (Figure 1). Basic clinical and pathological characteristics are shown in Table 2 and the samples flow chart is described in Figure 1. Analyzing normalized mRNA expression values of evaluated HER (ERBB) family marker genes and MKI67, lowest expression levels were observed for ERBB3 (Median 33) and ERBB4 (Median 32.7) and highest expression levels for ERBB2 (Median 35.9) and MKI67 (Median 35.6, Figure 2) using the two-way Mann-Whitney test. Measured Median Cq values of analyzed genes were between 27.4 (ERBB3; (28.3 ERBB2; 30.7 MKI67; 32.1 EGFR)) and 32.6 (ERBB4). Using Spearman's correlations between the analyzed genes weak negative correlations were found between EGFR (*ERBB1*) and* HER2 *(*ERBB2* Rho 0.18, *p* = 0.22) and moderate to high positive correlations between* HER4 *and* ERBB2 (HER2), ERBB3 (HER3)* and* ERBB2 (HER2), and ERBB4 (HER4)* and* ERBB3 *(*HER3, *Rho ranges from 0.44 to 0.64, *p* < 0.01 in all cases).

### 3.2. Association of Gene Expression with Patient and Tumor Characteristics

All biopsies (*n* = 52) irrespective of treatment modality showed no association between normalized mRNA expression and age (≤65 versus >65 years) or gender. However, higher* EGFR (ERBB1)* expression levels were associated with the risk for developing recurrence (median: 35.2 versus 34.7; *p* = 0.036) or death during follow-up (median: 35.1 versus 34.7; *p* = 0.038). Furthermore a trend was observed for higher* HER3* expression levels among patients still alive during follow-up (median: 33.2 versus 32.4; *p* = 0.083). No significant association of normalized gene expression was observed in relation to different treatment groups (5-fluorouracil versus capecitabine). Comparison of normalized gene expression in biopsies versus resected specimen in the adjuvant treatment group showed that all markers with the exception of* EGFR (ERBB1)* displayed higher expression levels in preoperative biopsies than in resection specimens (Table 3). Therefore solely pretreatment biopsies were analyzed with regard to DFS and OS.

### 3.3. Association of Gene Expression with Disease-Free and Overall Survival

**Figure 3:**
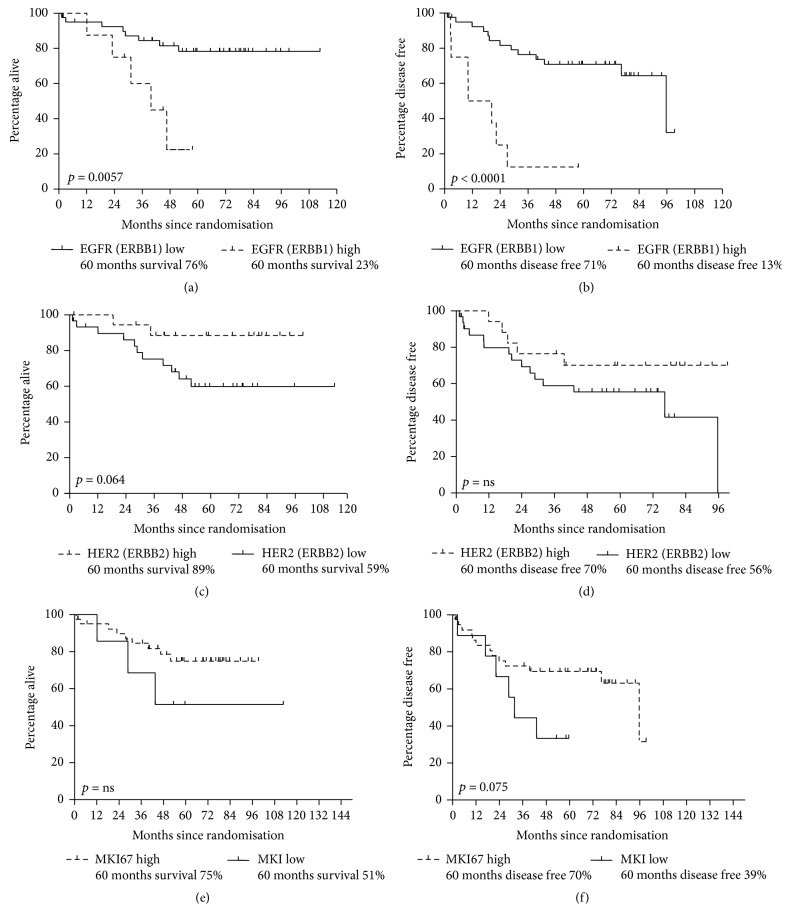
(a) Kaplan-Meier curve for overall survival (OS) according to the mRNA status of* EGFR (ERBB1)*. The predictive values for* EGFR (ERBB1) *high and low expression were estimated using the partitioning test. Five-year overall survival for low* EGFR (ERBB1) *expression was 76%, for high* EGFR *expression 60%; *p* = 0.0057. (b) Kaplan-Meier curve for disease-free survival (DFS) according to the mRNA status of* EGFR (ERBB1)*. The predictive values for* EGFR (ERBB1) *high and low expression were estimated using the partitioning test. Five-year disease-free survival for low* EGFR (ERBB1)* expression was 71%, for high* EGFR (ERBB1) *expression 13%; *p* < 0.0001. (c) Kaplan-Meier curve for overall survival (OS) according to the mRNA status of ERBB2 (HER2). The predictive values for* ERBB2 (HER2) *high and low expression were estimated using the partitioning test. Five-year overall survival for low HER2 (ERBB2) expression was 59%, for high* HER2 (ERBB2) *expression 89%; *p* = 0.064. (d) Kaplan-Meier curve for disease-free survival (DFS) according to the mRNA status of ERBB2 (HER2). The predictive values for* ERBB2 (HER2) *high and low expression were estimated using the partitioning test. Five-year overall survival for low HER2 (ERBB2) expression was 56%, for high* HER2 (ERBB2) *expression 70%; *p* = n.s. (e) Kaplan-Meier curve for overall survival (OS) according to the mRNA status of* MKI67*. The predictive values for* MKI67 *high and low expression were estimated using the partitioning test. Five-year overall survival for low* MKI67 *expression was 75%, for high* MKI67* expression 55%; *p* = n.s. (f) Kaplan-Meier curve for disease-free survival (DFS) according to the mRNA status of* MKI67*. The predictive values for* MKI67 *high and low expression were estimated using the partitioning test. Five-year overall survival for low* MKI67 *expression was 39%, for high* MKi67 *expression 70%; *p* = 0.075.

For the* EGFR (ERBB1)* mRNA values the evaluated cut-off (18% high expression) was prognostic for both OS and DFS, while for the* ERBB2, ERBB3, *and* MKI67* no significant prognostic effect was found in the examined cut-offs in terms of OS and DFS. Concerning* EGFR (ERBB1)*, 5/8 deaths (63%) and 7/8 relapses (88%, 6 distant metastasis) occurred in patients with high-expressing tumors compared to 8/41 deaths (20%) and 13/41 relapses (31%, 8 distant metastasis) in the low-expressing tumors. Patients with high mRNA expression of EGFR (*ERBB1*) had increased risk for death (HR = 4.75, 95% CI: 1.51 to 14.48, *p* = 0.0090) and increased risk for relapse (HR = 7.04, 95% CI: 2.6 to 18.84, *p* = 0.0003) compared to patients with low-expressing tumors. Kaplan-Meier curves for OS and DFS according to the mRNA status of EGFR (*ERBB1*) are displayed in Figures 3(a) and 3(b).

For* ERBB2 (HER2)* mRNA expression (38% high expression) a trend for a prognostic association was observed for OS (*p* = 0.064) and no correlation for DFS. Furthermore, patients with high* MKI67* mRNA expression (82% high expression) showed a trend for a reduced DFS using the Kaplan-Meier method (*p* = 0.075; Figures 3(c), 3(d), and 3(f)). Concerning* ERBB2 (HER2)*, 2/19 deaths (11%) occurred in patients with high-expressing tumors in comparison to 11/30 deaths (37%) in the low-expressing tumors. Patients with high mRNA expression of* ERBB2 (HER2)* had reduced risk for death (HR = 0.27, 95% CI: 0.041 to 0.99, *p* = 0.048) in the proportional hazard model compared to patients with high-expressing tumors. Kaplan-Meier curves for OS according to the mRNA status of* ERBB2 (HER2)* are shown in Figure 3(c).

With regard to* MKI67*, 13/39 relapses (33%) occurred in patients with high-expressing tumors in comparison to 6/9 relapses (67%) in the low-expressing tumors. Patients with high mRNA expression of* MKI67* had reduced risk for relapse (HR = 0.32, 95% CI: 0.13 to 0.88, *p* = 0.029) in the proportional hazard model compared to patients with low-expressing tumors when adjusting for treatment group. Kaplan-Meier curves for DFS and OS according to the mRNA status of* MKI67* are shown in Figures 3(e) and 3(f).

## 4. Discussion

No molecular markers have been described thus far to identify patients with LARC carrying a high risk for distant metastases. The aim of this study was to assess the potential prognostic value of ERBB family of receptors (*EGFR (ERBB1), ERBB2 (HER2), HER3 (ERBB3),* and* HER4 (ERBB4)* expression), as well as* MKI67* expression in patients with LARC treated at a single center with chemoradiotherapy based on capecitabine or fluorouracil within a phase 3 clinical trial. Expression of* EGFR (ERBB1)* was prognostic for both, OS and DFS, in this analysis.

The mRNA expression of the four HER family members has previously been assessed from a series of 100 locally advanced rectal cancers treated with radiotherapy or radiochemotherapy and showed an association for EGFR (ERBB1) and HER3 gene expression with development of distant metastases in LARC [19]. The current analysis confirmed that higher* EGFR (ERBB1)* expression levels were found more frequently among patients developing metastases. Patients with high mRNA expression of* EGFR (ERBB1)* also had an increased risk for death compared to patients with low-expressing tumors. In contrast,* ERBB3* expression was not prognostic in our analysis. However, both studies are not completely comparable as in the present study analysis for* ERBB1–4* and* MKI67* was based on a standardized RNA extraction method and uniformly staged and treated (5-FU based chemoradiotherapy) patients were investigated in our series. Moreover, Ho-Pun-Cheung and colleagues used two different RNA extraction protocols (TRIzol and Qiagen Columns). These differences may explain the variability in the results. Comparably, both studies used* pretreatment* specimen and RNA based PCR expression. EGFR (ERBB1) immunohistochemistry and RT-qPCR showed a good overall concordance of 81% in breast cancer patients as described recently [21]. This suggests related mRNA gene expression and protein levels for EGFR (ERBB1). Pretreatment tumor biopsies were also investigated in another study comprising 40 patients with LARC [24]. Responders to neoadjuvant 5-fluorouracil-based chemoradiotherapy (patients with significant tumor regression) showed significantly lower* EGFR (ERBB1)* gene expression levels than nonresponders (patients with insignificant tumor regression). The 3-year DFS rates of the patients with lower gene expression levels of* EGFR (ERBB1)* were significantly higher (90%) than those of the patients with higher gene expression levels (70%) (*p* = 0.003). These data are in line with the results of our current investigation where patients with high mRNA expression of* EGFR (ERBB1) *had increased risk for relapse and death compared to patients with low-expressing tumors treated with capecitabine or 5-FU. This provides evidence for the use of EGFR targeted therapies in patients with high expression of EGFR treated with capecitabine or 5-FU.

The predictive value of MKI67 was investigated in a rectal cancer patient cohort treated with neoadjuvant 5-FU based chemoradiotherapy [25]. A small set of formalin-fixed, paraffin-embedded pretreatment tumor biopsies and posttherapeutical resection specimens were studied by immunohistochemistry. The results were compared with histopathological tumor regression according to a standardized semiquantitative scoring system. Responders (patients with high tumor regression) showed a significantly lower MKI67 expression than nonresponders in the pretherapeutical tumor biopsies (81.2% versus 16.7%; *p* < 0.05) as well as in the posttherapeutical resection specimens (75.8% versus 14.3%; *p* < 0.01). Despite obvious differences regarding the aims and methods of both investigations it is surprising that the results appear to be diametrically opposed. Clearly, the number of pretreatment tumor biopsies in the cited publication was relatively small (*n* = 22). As described in the Results, sampling methods (i.e., biopsies versus resection) may explain differences in gene expression levels. However, this does not explain the difference between both data sets. In primary breast cancer, for instance, high* MKI67* mRNA expression measured by RT-qPCR is predictive for achieving pathological complete remission (pCR) to neoadjuvant chemotherapy [26]. RT-qPCR was superior to MKi67 determined by immunohistochemistry [27]. Even though breast cancer and rectal cancer have different biologies the applied techniques, immunohistochemistry versus mRNA expression analysis by RT-qPCR, may be in part responsible for the observed differences.

With regard to* HER2/ERBB2* we found a trend for a reduced risk of death for HER2 high-expressing tumors. The role of* HER2*/*ERBB2* expression in colorectal cancer is very controversial. In a series of 1645 patients using immunohistochemistry and chromogenic in situ hybridization (CISH) with standard protocols an overall positivity rate of 1.6% in CRC patients was described [28]. Contrarily, using immunohistochemistry (IHC) scoring and detection of silver in situ hybridization amplification (SISH) HER2 status was determined in patients with rectal cancer (*n* = 264). Tumors with an IHC score of 3 or SISH ratios of ≥2.0 were classified HER2 positive. HER2 status was found to be positive in 12.4% of patients with pretreatment biopsies. In this analysis, patients with HER2 positivity showed a trend for better DFS and a significant benefit in cancer-specific survival. Five-year survival rate was 96.0% for patients with HER2 positive tumors (versus 80.0% for HER2 negative tumors) [29]. Recently, an Italian group has proposed specific criteria for HER2 positivity in colorectal cancer [30]. The same group has evaluated anti-HER2 treatment (lapatinib + trastuzumab) within the so-called HERACLES trial in HER2 positive colorectal cancer patients as last line treatment and found significant antitumor activity [31]. Taken together, our results support the findings of Conradi and coworkers postulating a positive prognostic effect of HER2 gene expression. However, further investigations regarding the optimal method of defining HER2 positivity are needed, particularly since targeting HER2 has resulted in excellent efficacy data even in heavily pretreated patients in the HERACLES trial.

With the exception of EGFR (*ERBB1*), normalized gene expression was higher in biopsies than in resection specimens. This difference could be a result of the effect of ischemia. Surgical procedures significantly affect the expression of genes in colorectal cancer tissue seen as a significant difference in the molecular composition of tissue specimens collected after tumor resection compared to specimens collected via colonoscopy before tumor resection [32]. In the present analysis, normalized values were also affected. This clearly implies that ischemia alters gene expression in a gene-specific manner. This was also shown by Liu and colleagues using microarray and PCR techniques in kidney cancer [33] which clearly underlines the importance of strict standardization and documentation of preanalytical factors. Therefore, only pretreatment biopsies were evaluated in this study to exclude an influence of sampling method on gene expression.

## 5. Conclusion

The key findings of our analysis of ERBB family in patients with locally advanced rectal cancer undergoing 5-fluorouracil based chemoradiotherapy are as follows: (i)* EGFR (ERBB1)* mRNA levels had a prognostic significance for DFS as well as for OS. (ii) Differences between pretreatment biopsies and resected specimen underline the importance of standardizing preanalytical procedures in biomarker studies. (iii) High MKI67 mRNA expression correlated by trend with DFS. (iv) A relevant proportion of patients had HER2 positive tumors and therefore a better prognosis. On the other hand, HER2 positivity may offer the possibility for studying trastuzumab-based treatment in this disease.

## Figures and Tables

**Table 1:** Primer/probe sets used for amplification of the target and reference genes.

Gene	Oligo ID	Oligo sequences 5′-3′	Label probe	Amplicon length (nts)	Assay location	PCR efficacy in %
*MKI67*	Forward	CGAGACGCCTGGTTACTATCAA	CY5-BHQ2	108	c. ex 2-3 NM_002417	98
	Reverse	GGATACGGATGTCACATTCAATACC				
	Probe	ACGGTCCCCACTTTCCCCTGAGC				

*CALM2*	Forward	GAGCGAGCTGAGTGGTTGTG	YY-BHQ1	72	c. ex 1-2 NM_001743	99.3
	Reverse	AGTCAGTTGGTCAGCCATGCT				
	Probe	TCGCGTCTCGGAAACCGGTAGC				

*EGFR (ERBB1)*	Forward	CGCAAGTGTAAGAAGTGCGAA	FAM-BHQ1	93	NM_201283	89.1
	Reverse	CGTAGCATTTATGGAGAGTGAGTCT				
	Probe	CCTTGCCGCAAAGTGTGTAACGGAAT				

*ERBB2 (HER2)*	Forward	TCTGGACGTGCCAGTGTGAA	CY5-BHQ2	61	M11730_gen	97.2
	Reverse	CCTGCTCCCTGAGGACACAT				
	Probe	AGGCCAAGTCCGCAGAAGCCCT				

*ERBB3 (HER3)*	Forward	CGGTTATGTCATGCCAGATACAC	ROX-BHQ2	81	NM_001982	96
	Reverse	GAACTGAGACCCACTGAAGAAAGG				
	Probe	CTCAAAGGTACTCCCTCCTCCCGGG				

*ERBB4 (HER4)*	Forward	GAGGCTGCTCAGGACCTAAGG	ATTO-BHQ1	75	NM_005235	92
	Reverse	GAGTAACACATGCTCCACTGTCATT				
	Probe	CACAGACTGCTTTGCCTGCATGAATTTC				

CY5 = cyanine dye 5, BHQ = black hole quencher; FAM = 6-carboxyfluorescein; ROX = carboxy-X-rhodamine; ATTO = fluorescent dye (*ATTO*-TEC GmbH, Siegen, Germany).

**Table 2:** Patients' characteristics.

	Neoadjuvant treatment	Adjuvant treatment
*N* (patients)	30	56

Age (years), median [range]	67 [47–83]	62 [44–75]

Gender, *n* (%)		
Male	20 (67)	36 (64)
Female	10 (33)	20 (36)

5-Fluorouracil, *n* (%)	15 (50)	27 (48)

Capecitabine, *n* (%)	15 (50)	29 (52)

Recurrence during follow-up, *n* (%)		
Local recurrence	1 (3)	3 (5)
Metastasis	9 (30)	15 (27)

**Table 3:** Comparison of normalized gene expression values (40-ΔCq) in biopsies versus resected specimen in the adjuvant treatment group. Significant lower expression in resected specimen was observed for HER2–HER4 and MKI67 (n.s.: not significant).

Gene	Biopsy (*n* = 22)	Resected specimen (*n* = 54)	*p* value
	Gene expression median (range)	Gene expression median (range)	
EGFR (ERBB1, HER1)	34.8 (32.9–36.1)	34.6 (33.2–36.1)	n.s.
HER2 (ERBB2)	36.0 (34.6–37.2)	35.4 (34.3–36.5)	0.039
HER3 (ERBB3)	33.1 (31.9–34.5)	32.1 (30.6–33.4)	<0.001
HER4 (ERBB4)	32.9 (29.8–34.5)	31.8 (29.1–34.3)	<0.001
MKI67	35.4 (33.5–36.8)	35.1 (30.4–36.4)	0.02
